# Assess, Plan, Do, Evaluate, and Report: Iterative Cycle to Remove Academic Control of a Community-Based Physical Activity Program

**DOI:** 10.5888/pcd18.200513

**Published:** 2021-04-08

**Authors:** Samantha M. Harden, Laura E. Balis, Thomas Strayer, Meghan L. Wilson

**Affiliations:** 1 Department of Human Nutrition, Foods, and Exercise, Virginia Polytechnic Institute and State University, Blacksburg, Virginia; 2 University of Wyoming Extension, Lander, Wyoming; 3 Vanderbilt University Medical Center, Center for Quality Aging, Nashville, Tennessee; 4 Bluefield College, Department of Biology, Bluefield, Virginia; 5 Edward Via College of Osteopathic Medicine, Department of Preventive Medicine and Public Health, Blacksburg, Virginia

## Abstract

**Purpose and Objectives:**

Responsive methods and measures are needed to bridge research to practice and address public health issues, such as older adults’ need for multicomponent physical activity. The objective of this study was to detail the longitudinal, quasi-experimental work that spans 5 years to describe outcomes across RE-AIM (reach, effectiveness, adoption, implementation, and maintenance) dimensions of integrating a physical activity intervention for older adults into the Cooperative Extension System through the assess, plan, do, evaluate, report (APDER) cycle.

**Intervention Approach:**

The participant-level intervention is Lifelong Improvements through Fitness Together (LIFT), an 8-week, group dynamics-based, strength-training program with 16 in-person sessions. The implementation intervention applies the iterative APDER cycle based on feedback for each dimension of RE-AIM. Each year, the APDER cycle was used to embed data collection procedures at the instructor and participant level to reveal the next evolution of the program.

**Evaluation Methods:**

Each evolution of LIFT was measured through a pretest and posttest quasi-experimental design. Data were collected on each RE-AIM dimension through participant surveys and functional fitness assessments, number and representativeness of trainees, and process evaluation.

**Results:**

Overall, LIFT was expanded to 4 states with 275 instructors, reaching 816 older adults; consistently improved functional fitness outcome measures; demonstrated strong program adherence; and was seen as feasible and enjoyable by instructors and participants. LIFT is now undergoing adaptations for virtual delivery as well as updating the exercise protocol to introduce yoga postures that target flexibility and balance.

**Implications for Public Health:**

Overall, ongoing adaptations were necessary to ensure the program continued to fit the mission, values, and resources of the delivery system. Public health implications to support the need for ongoing adaptation include embedding pragmatic measures of adaptations and RE-AIM into standard evaluation pathways and using iterative APDER cycles.

**Summary**

**What is already known on this topic?**

Multicomponent physical activity interventions are needed to increase the proportion of older adults meeting the Physical Activity Guidelines for Americans.

**What is added by this report?**

Because a one-size-fits-all approach has shown to thwart the translation of evidence-based programs into practice, a focus on intervention core elements and adaptability has emerged.

**What are the implications for public health practice?**

Based on the needs of different audiences, researchers are called to train and support delivery staff in their ability to adapt, implement, and evaluate community-based efforts.

## Introduction

The lofty goal of integrating evidence-based interventions into community settings—and all the models, measures, and methods available for this task—leaves one wondering if this effort is a service for improving the lives of participants. A disconnect exists between the outcomes valued by the systems that house researchers and those of community stakeholders. Academics are pushed within a publish-or-perish cycle (1), whereas community partners need trust, autonomy, incentives, and effects (2,3). Despite these different system-level measures of time and effort, research recommends that academic and community partners work together to understand infrastructures (resources, staff, values), core elements of an intervention, and ways to increase the likelihood of health equity and program sustainability (4). Taken together, participatory approaches that identify, adapt, and deliver programming that meet the needs of the community ensure a balance between academia and community to ultimately reduce translational delays and improve public health. Traditional implementation science methods, however, have not resulted in a sustained delivery of evidence-based programs in the real world (5). New approaches are needed to speed translation from research to practice and integrate priorities of both systems (5).

To address this need for pragmatism, generalizability, comprehensive planning, and evaluation (6), the RE-AIM framework has been used in several settings and populations for the last 20 years (7). RE-AIM stands for reach (who), effectiveness (what impacts), adoption (who and where is it delivered), implementation (how well it is delivered and at what cost), and maintenance (behavior change maintenance and institutionalization of the intervention) (8). These are key variables that delivery staff and stakeholders use in choosing an intervention, particularly considering needs for tailoring or adaptation and evaluation (9–11). In many cases (12), RE-AIM has been applied in pragmatic, real-world contexts to guide decision making with limited extramural funding, indicating the framework’s ability to be useful whether the program is a service or a study. Furthermore, to account for the dynamic nature of delivering interventions in the real world, RE-AIM can be applied before, during, and after intervention initiation, through an assess, plan, do, evaluate, report (APDER) cycle (11).

These responsive methods and measures can be used to address one of the most prominent public health issues affecting the health of the aging population: the need for social engagement and multicomponent physical activity. Low physical activity compliance indicates that efficacious exercise programs for older adults are not readily translated into sustained practice (13). Although extensive literature is available to support community-delivered physical activity programs for older adults in settings such as the YMCA, less is known about targeting the federally funded Cooperative Extension System (14). The Cooperative Extension System is ideal structure for dissemination, as it is available in all states and territories and has county-based agents who are trained in evidence-based interventions by university-based specialists (14,15). Embedding robust outcome evaluation, however, has been challenging for Extension professionals, especially because the system values a variety of data sources and types (16). Finally, rather than adapt existing Extension interventions, programs are duplicated (rebranded) and not collated (matched) for national effect (17).

## Purpose and Objectives

Few studies have detailed the long-term process of delivering interventions in the real world, including using an iterative process to document and respond to adaptations through a research practice–partnership (18). The purpose of this study, therefore, was to document program adaptations that occurred as a result of our implementation strategy: iterative APDER cycles used to improve an older adult physical activity program, the Lifelong Improvements through Fitness Together (LIFT) program, from an efficacy trial to an ongoing, community-based program. Although information on the effectiveness and maintenance of LIFT itself is used to provide a holistic picture of the implementation evaluation, it is not the focus of our work here. As articulated by earlier research (19), this implementation study was primarily focused on the “stuff we do to help people do the thing” (ie, the APDER cycle) rather than “the thing” (ie, the LIFT program). The primary outcomes were adaptations made to 1) LIFT data collection protocols based on the RE-AIM framework and 2) LIFT components (setting, target audience, mode of delivery, cultural adaptations, core components) based the Adaptome (19).

The APDER (assess, plan, do, evaluate, report) process was collaboratively conducted by a university-based exercise specialist, graduate research assistants, and the Physical Activity Leadership Team (PALT; county-based agents housed within Virginia Tech serving Virginia Cooperative Extension) (18). To support the iterative process of understanding programming needs, adaptations, and evaluation (competency and capacity), all members of PALT met annually to develop program evaluation reports based on the APDER cycle. For example, when reach data showed low representativeness of non-White participants or when implementation process evaluation data were not being returned, the integrated research-practice team was able to adjust as needed and capture why, what, and how adaptations were made. One response to low racial/ethnic diversity among participants was PALT members serving as program champions and cohosting training to encourage their district colleagues to deliver the program (2,20–22). More racial diversity among LIFT instructors led to greater diversity in LIFT participants. In alignment with the integrated research-practice approach, research and practice needs were equally valued (18), and decisions were made by consensus.

LIFT is an 8-week, group dynamics-based strength-training program that has 16 in-person sessions (23). During the 16 sessions, participants follow a similar guide for group dynamics strategies that have worked with a number of populations (24). In weeks 7 and 8 (the final sessions) group strategies focus on relapse prevention by preparing for program termination and long-term behavior change. The sequence for LIFT’s 8 recommended full-body exercises (25) is wide-leg squat, standing leg curl, seated knee extension, side-hip raise, biceps curl, overhead press, seated bent-over rows, and toe stand. The focus of this sequence is on the entire body and provides an opportunity for participants to stand and sit, improving functional fitness in the interim of exercise. The exercises take approximately 50 minutes to complete, allowing time for the agents to facilitate a group dynamics-based warm-up as well as cool-down stretching within the 60-minute class.

Repetition of LIFT exercises in each class (ie, 3 sets of about 10 repetitions) and across the 8 weeks allows participants to become familiar with the routine over time. Participants are asked to engage in aerobic activity to reach a minimum of 150 minutes of moderate-to-vigorous aerobic activity per week outside LIFT class time. Participants who were previously inactive, however, are encouraged to move more as they progress to meet recommendations. Instructors facilitate goal setting, feedback, and self-monitoring to increase aerobic activity levels. Ultimately, core elements of LIFT provide opportunities for group engagement (friendly competition, interaction, problem solving), experiential learning for strength training exercises (repetition), and promotion of behavior change strategies (goal setting, self-monitoring).

LIFT was tested in 1 state system before its national launch. Based on the success of the program to retain participants, objectively measured functional fitness improvements, and ease and enjoyment of program delivery, PALT adopted LIFT as a statewide program (23,26). Only the few agents who delivered the program in the first year, however, had these successes with the program (Figure).

**Figure:**
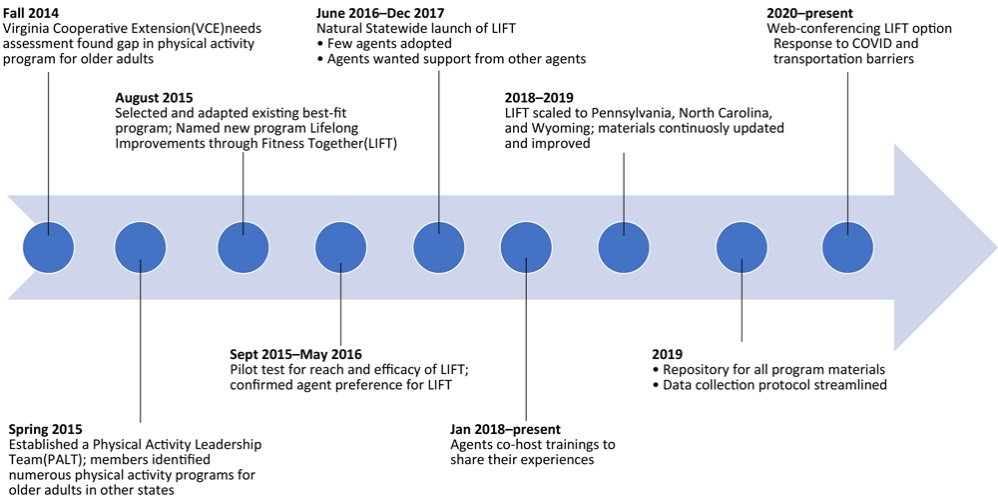
Timeline from 2014 to 2021 and beyond showing progressive milestones for Physical Activity Leadership Team (PALT) adopting Lifelong Improvements through Fitness Together (LIFT) as a statewide program.

In a complementary investigation, a survey was distributed and semistructured interviews were conducted to determine why educators who attended LIFT training chose to deliver the program or not. Intent to deliver LIFT was based on the Stages of Change (27) and a 5-point Likert scale. We found no significant difference between deliverers and nondeliverers in intent to deliver (mean [SD], 3.8 [1.1]). Training satisfaction was based on a 60-point adapted training satisfaction scale (28). Both deliverers and nondeliverers reported high training satisfaction in objectives and content, methods and training context, and the usefulness and overall rating (51.8 of 60) immediately posttraining. Posttraining, nondeliverers were significantly more likely to want more training on delivering physical activity interventions (*P* = .045), to feel that the physical activity interventions are not part of their job (*P* = .04), and to report that they are not physically active, so do not feel comfortable delivering a physical activity intervention (*P* = .001). Deliverers were significantly more likely (*P* = .02) to be preparing to deliver a physical activity intervention than their nondeliverer counterparts.

Overall, deliverers and nondeliverers reported high training satisfaction, the need for structured peer support, and a desire for ongoing training. Frequently reported barriers to implementation included the complexity of the intervention, cost of equipment, and low self-efficacy in physical activity and physical activity delivery. Frequently reported facilitators to adoption included assistance from the research team and other Cooperative Extension staff to reduce delivery burden, positive perceptions of pragmatic fit of the intervention, and positive perceptions of the effectiveness of the intervention. Nondeliverers were more likely to report barriers than facilitators, although deliverers reported both barriers and facilitators.

Based on older adult efficacy and agent feedback and enthusiasm, a standardized training protocol was developed and vetted through PALT. The training program involves detailed explanation of underlying program principles (eg, group dynamics-based activities) and experiential learning to practice the exercises and fitness assessments in small groups. After the 8-hour, in-person training, ongoing assistance and support was provided by use of web conferencing. This assistance aligned with Cooperative Extension’s standards of ongoing implementation for peer education, program support, and specialists’ availability.

Older adults (aged ≥65 y) with a working comprehension of English (for consent, safety cues, and program evaluation) and residents of participating counties were eligible to join the program. Cooperative Extension provides open-access programming to all Americans as part of its civil rights mandate, including, for example, programs designed for Hispanic audiences (17,29). More work is needed, however, to translate LIFT and other Cooperative Extension programs for non-English speaking audiences (28). As this was a real-world effectiveness trial based on a community program, no further inclusion or exclusion criteria were used, and all LIFT program participants were invited to be research participants.

Participants completed the Physical Activity Readiness Questionnaire for Everyone, which was developed for inclusion of older adults who might benefit from participating in physical activity, but who have a managed chronic condition. Agents recruited cohorts of older adults from within the counties they serve. Each agent used a variety of methods for recruitment including targeted mailings, newsletters and newspapers, word of mouth, flyers, and presentations at existing programs. Agents also leveraged existing community ties to recruit from local retirement and assisted living facilities. The Virginia Tech Institutional Review Board approved the entirety of this work.

## Evaluation Methods

Reach, Effectiveness, Adoption, Implementation, and Maintenance (RE-AIM) data collection methods and adaptations to LIFT core components were determined through annual reviews of LIFT program evaluation data, captured in Cooperative Extension annual reports. These reports were based on data acquired through a pragmatic, quasi-experimental, mixed-methods evaluation protocol implemented from 2016 through 2020. Each year, research team members collated individual-level outcome data (ie, reach, effectiveness) as well as number and representativeness of trainees and process evaluation (ie, adoption, implementation data). RE-AIM annual reports were developed based on national requirements of Cooperative Extension impact statements, which discuss relevance, response, and results. Impact statements are a combination of quantitative and qualitative data. More specific data analysis, based on measurement, are reported in primary outcome articles elsewhere (23,26,30).

## Results

We present adaptations based on RE-AIM dimensions (Table 1) and a rapid deductive analysis of Adaptome categories, based on the survey and email correspondence with state administrators and adaptations to core components by state (Table 2). PALT meeting notes with key outcomes and decision pathways are presented longitudinally to document the APDER process and adaptations made over time (Figure).

**Table 1:** Fundamental Evaluation Protocol for RE-AIM Dimensions and Measures

Dimension	Outcome Measures	APDER Feature and Notes
**Reach**		
Number, proportion, and representativeness of participants	Number, proportion, and representativeness of LIFT participants assessed via survey	• Individual-level sociodemographic data are required for reporting Cooperative Extension efforts. These survey items were initiated in 2015, continuing since then in each state • State administrators determine representative data; LIFT participant sociodemographic information can be compared to the full state census data or compared to the counties from which the participants were recruited
**Effectiveness**		
Effect on primary outcomes, quality of life, and unintended consequences	Objectively measured functional fitness assessment and survey for self-report items of interest (social connection, physical activity behaviors)	• Educators and volunteers found it cumbersome to administer the Rikli and Jones functional fitness assessment (31) plus other assessments. For virtual adaptations, educators can allow participants to self-report functional fitness outcomes • Self-reported survey items changed over time to align with research questions, survey duration, or outcomes of interest; therefore, summary and comparisons across years is not possible, nor perceived as relevant by PALT
**Adoption**		
Number, proportion, and representativeness of settings and staff who deliver the intervention	• Number, proportion, and representativeness were measured for Cooperative Extension health educators and community partners who implement LIFT • What steps were taken in delivering LIFT	LIFT training included pretraining and posttraining surveys to assess instructor sociodemographic characteristics with intent to deliver LIFT, and program content (ie, teach-back).
**Implementation**		
Degree that intervention was delivered as intended	Process evaluation checklists for every LIFT session	• Process evaluation was available in paper and pencil or online • Low instructor compliance limited interpretation • State administrators surveyed to assess state adherence to LIFT principles and delivery
**Maintenance (system level)**		
Extent to which delivery and implementation are sustained over time	Number of years LIFT is delivered in the county or state	Monitored via LIFT program records by the LIFT program manager. In 2021, a protocol to follow up with all trained staff will be launched.

Abbreviations: APDER, assess, plan, do, evaluate, report; LIFT, Lifelong Improvements through Fitness Together; PALT, Physical Activity Leadership Team; RE-AIM, reach, effectiveness, adoption, implementation, and maintenance.

**Table 2:** Summary of State Adaptations to LIFT Program State

Virginia	Wyoming	Pennsylvania	North Carolina
**Setting**			
Delivered in a variety of facilities including YMCA, schools, libraries, churches, and through Parks and Recreation	Discontinued. In 2017, there were 6 nutrition educators; by 2020, only 2 across the state. Educators and administrators did not have resources to support delivery	Delivered by trained instructors across the county through Cooperative Extension, with a standardized fee	Delivered predominantly online due to COVID-19
**Selected audience**			
Adults ages ≥65 y who are inactive or insufficiently active	NA	Adults ages ≥65 y, fee-based program (with tuition options for lower incomes); predominantly female; many participants continue program participation throughout the year (ie, not new participants every session)	Expanded to those aged <65 y; “During our initial discussions our target audience was defined as limited-resource individuals of any age”
**Mode of delivery**			
Virtual delivery allows more modes available for in 2020	NA	Predominantly in person; exploring virtual and in person with masks during COVID-19 public health restrictions	Added a Facebook Live session delivery option during COVID-19
**Cultural adaptations—**Agents expressed concern for LIFT imagery, including White-only and lean-bodied older adult models. More representation in LIFT materials is needed for all states.			
**Core components**			
Added yoga asanas in 2020 to improve flexibility and balance outcomes	NA	Added some advanced Strong Women/Strong Bones exercises (indicated on the process evaluation form); added state’s nutrition education handouts (ie, beyond LIFT’s embedded nutrition messaging)	NA

Abbreviation: LIFT, Lifelong Improvements through Fitness Together; NA, not available.

### 2016–2018

From 2016 through 2018, LIFT was evaluated in its original state, reaching 258 participants using 21 trained educators. In 2016, 139 older adults participated; in 2017, 63 participated; and 56 participated in 2018. Participants were predominantly White (70%) and aged 73, with a body mass index of 31. Overall, participants provided positive feedback about LIFT. Data were used in annual impact statements required by the state system that include program relevance, response, and results. Reach, effect (functional fitness), and illustrative quotes were the data used to drive decision making.

### 2018-2019

Program results (2016–2018) were considered strong for a Cooperative Extension program and from 2018 through 2019, 3 additional states were trained for LIFT: Wyoming, Pennsylvania, and North Carolina. Through these partnerships, an additional 269 LIFT instructors (agents and community partners) were trained: 24 in North Carolina, 13 in Wyoming, 114 in Pennsylvania, and 83 additional instructors in Virginia (Table 2).

### 2020 and beyond

Although functional fitness assessment (31) remained the primary outcome measure, participant survey items were adapted over time. For example, the original surveys were double-sided, multiple-paged, and time-consuming. Members of PALT and other instructors suggested condensing the text of the surveys to 1 page, front and back. Font size, but not content, changed on the survey. The length of the Physical Activity Group Environment Questionnaire (32) had been perceived as a participant burden; therefore, PALT opted for a shorter social network scale instead. In addition, and to aid in open-access, a program repository became available at www.parcilab.org/lift. The repository is updated as needed and contains all paper and electronic versions of data collection tools, training slide decks, and all program materials.

To produce an annual national impact statement, all state LIFT coordinators were asked to complete a 5-minute report — based on Adaptome (20) and RE-AIM (7) — in October of each year. This includes 17 items on a 5-point Likert scale within RE-AIM dimensions. Open-ended questions in the report are based on Adaptome categories (19) and inquire whether adaptations were made, such as “Have you made any adaptations for who can deliver the program?” Pennsylvania and North Carolina administrators had positive perceptions across each dimension of RE-AIM in their annual report; however, North Carolina administrators shared that they did not collect outcome data at 6 months. North Carolina staff were trained just before the COVID-19 pandemic and were delivering the intervention online. Wyoming did not complete the survey because LIFT was discontinued (Table 3).

**Table 3:** Administrator Perceptions of RE-AIM, 2020

RE-AIM Dimensions	Quantifiable Scale (1–5 Points)	Pennsylvania	North Carolina
Reach	Overall, participants were representative of older adults in our catchment area.	Agree	Agree
	Our recruitment strategies ensured that all eligible people felt supported to attend.	Agree	Strongly agree
	Costs of recruitment were embedded within usual practice.	Agree	Neither agree nor disagree
Effectiveness	Our participants had measurable functional fitness improvements.	Agree	Agree
	Our participants were more socially connected.	Agree	Strongly agree
Adoption	A large proportion of eligible instructors were trained on LIFT (agents, volunteers, educators).	Agree	Strongly agree
	Trained LIFT instructors were representative of our staff (years working with Cooperative Extension, age, race, etc.).	Agree	Strongly agree
	Training costs fit within our resources.	Agree	Strongly agree
Implementation	Our LIFT instructors felt confident delivering the core elements of LIFT.	Agree	Strongly agree
	Our instructors knew what an appropriate adaptation would be.	Agree	Strongly agree
	Our instructors reported adaptations.	Agree	Agree
	Delivery time for LIFT met my expectations.	Agree	Strongly agree
Maintenance/individual level	Participants will continue with an exercise routine.	Agree	Agree
	Participants have sustainable fitness.	Agree	Agree
	We measured long-term outcomes (at 6 months).	Agree	Disagree
Maintenance/organizational level	We intend to deliver LIFT in the future.	Agree	Strongly agree
	We have financial support to keep LIFT running.	Agree	Strongly agree

Abbreviation: LIFT, Lifelong Improvements through Fitness Together; RE-AIM, reach, effectiveness, adoption, implementation, and maintenance.

As with many other public health interventions, LIFT was adapted to virtual delivery in response to COVID-19. In summer 2020, a pilot project to examine the feasibility and effects of delivering LIFT by web conference was conducted through the Virginia Cooperative Extension. The project resulted in 11 participants with a weekly attendance average of (mean [SD], 4.7, [1.4]) participants. Through process evaluation, autoethnographic field notes, and participant tracking during the program, we detected that group dynamics strategies needed adaptation and that participants facilitated discussion by using audio and video. We anticipate that when in-person rapport is challenging, online LIFT delivery will encourage use of audio and video for additional contact with the instructor outside-of-class through social media posts, emails, and optional telephone calls. (Table 3).

## Implications for Public Health

Testing the adapted and newly packaged LIFT program took 5 years, a substantial decrease from the 17- to 24-year lag time for translation of research to practice (33). Overall, we found that ongoing adaptations at the educator and state levels were necessary to ensure the program continues to fit the mission, values, and resources of the system (34). This implementation evaluation has 4 primary public health implications.

First, we propose pragmatic measures of adaptations and RE-AIM that can be embedded within the standard evaluation pathway (6,8,12). Although, like many inner- and outer-setting construct measurements (35), the RE-AIM scale here was not validated but it did capture the information needed to determine if additional training or support was needed to integrate LIFT in new state systems. In addition to administrator perceptions of LIFT, LIFT has 2 key individual-level measurements: the self-report questionnaire and the functional fitness assessment. Administrators and instructors can choose which data are important to their partners and assess accordingly (10,11,36).

Second, we acknowledge the importance of the iterative cycle of assess, plan, do, evaluate, report and the nonlinear timeline (37); a full-scale efficacy trial for each adaptation is not feasible. Explicitly, efficacy trials for each adaptation are not necessary if the adaptation does not threaten program outcomes (eg, reach, effectiveness, fidelity). In fact, intervention developers should assume adaptations will occur and provide guidance for making appropriate adaptations (4,19,34,38). For example, materials for recruitment might require translation into other languages or literacy levels to better reach audiences across various ethnic groups and educational backgrounds. Additionally, if delivery agents prefer to deliver the program with music to increase the enjoyment of the activities, that would not negatively affect the functional fitness outcomes and might improve agent and participant enjoyment and therefore improve retention. Yet, researchers largely continue to retest intervention effects, leading to over-duplication of interventions and the loss of resources (39). For example, more than 20 different exercise programs exist for older adults in the Cooperative Extension System that are primarily based on Strong Women, Strong Bones (Strong Women) (17). With those programs, however, adaptations have occurred, data collection has halted, and Cooperative Extensions’ collective influence on physical activity of older adults is largely unknown (17). Cooperative Extension represents an implementation laboratory where we can study relatively stable inner and outer contexts and intervention updates (40). Our work, therefore, focuses on the importance of modifying interventions and disseminating information, so that all audiences have access to relevant information that informs decision-making processes for training, delivery, and participation at the administrator, instructor, and participant levels.

Third, we aim to remove academic control of a community-based physical activity program. We do this, in part, by providing an open-access program repository that includes materials on how to be a state administrator, how to provide training, and how to deliver and evaluate LIFT. This access is unique because 1) many evidence-based program repositories exist, but practitioners cannot always readily download materials to deliver the intervention (41); 2) many exercise programs for older adults require participants to pay a fee, which is a system-level barrier (42); and 3) Cooperative Extension professionals want relevant program information on-demand (43).

Finally, intervention costs are often a barrier to increasing the scale of a program (44). The open-access repository, therefore, aims to put the control into the hands of instructors and state administrators to ensure more people, particularly those representing socioeconomically disadvantaged communities, can offer the program. Costs considered instructor delivery time, participant travel, time for recruitment, equipment (whether individually purchased or provided by the county), and evaluation. In other older adult physical activity programs, training costs $250 per instructor (45). In some states, costs prohibited training new staff, therefore, state systems either 1) were unable to train new agents and maintain the program in the system or 2) developed their own programs that could be delivered at no additional cost. For example, in Wyoming, Cooperative Extension adopted Strong Women but did not maintain it. Two retired agents and a senior center, however, continued to deliver Strong Women in their communities and continued the program without Cooperative Extension involvement. Although delivery resulted in continued opportunities for older adults to engage in physical activity over the years, drift from the core components of the original program occurred (eg, inclusion of strength training exercises that did not appropriately target major muscle groups). When a new agent learned of LIFT and offered training to those delivering Strong Women, there was initial interest from other agents and community partners. After integrating and testing LIFT within the system, however, the agent left the system and the community partners went back to delivering Strong Women (ie, not sustaining the group dynamics, aerobic activity, or nutrition education components of the LIFT program). Additionally, although other agents expressed interest in delivering LIFT in Wyoming, only 2 agents attended training and only 1 agent delivered LIFT (30). Work is needed to better support community partners who have the time and ability to deliver physical activity programs. When agent positions become vacant, state-level specialists could continue to support community partners through training and curriculum updates to promote high-fidelity delivery of evidence-based programs.

As another example, agents in another state were trained in and delivered Strong Women, but the cost was prohibitive and a similar program, Extension Get Fit, was developed. Extension Get Fit was originally based on the same core exercises as Strong Women. However, program drift occurred over the years until agents and state-level staff were unclear as to the purpose of the program, the primary audience to focus on (ie, older adults vs adults of any age), or intended outcomes (eg, weight loss vs functional fitness). As the primary outcome of the program, functional fitness test results were reported as indicators in required national-level reports. Fewer participants improved in the aerobic endurance and agility portions of the functional fitness test than in the strength training components, likely because an aerobic warm-up was not consistently included as part of the Extension Get Fit program. Rather than incorporate aerobic activity as part of Extension Get Fit, as was incorporated into LIFT, staff created an additional circuit-training program that included aerobic activity. Participants also expressed interest in yoga and flexibility, and similarly, instead of incorporating a flexibility component into Extension Get Fit, another program focusing on chair yoga was added. The state system was ultimately supporting 3 separate programming efforts. County residents chose among the programs and did not receive an evidence-based program that included the comprehensive functional fitness components of strength training, aerobic activity, and flexibility. Multicomponent programs align with the national physical activity recommendations for older adults (ages ≥65 y) and have been shown to be more effective at improving physical activity outcomes (46).

Our study has limitations. First, all of the studies (ie, evolutions) discussed and designed were quasi-experimental, meaning that no randomization or causation could be explored. Second, as with any community work, representativeness and recruitment are limitations, as efforts to recruit undergo continuous improvement to reach intended audiences (7). The studies mentioned in this work attempted to nullify the lack of reach and effectiveness data by monitoring community needs assessments and demographic data to reach those that would benefit most from these interventions. Finally, the pragmatic nature of this study led to missing data across several levels. Intervention delivery staff and research staff made every effort to complete follow-up time points, as indicated by the approving institutional review board protocols. Empirically established reasons for missing data are unknown; however, anecdotally, agents shared that because LIFT is an open-access, community-based program, it is not seen as a research initiative. Therefore, participants do not feel obligated (or compensated) to provide data.

Communities desire interventions that are easy to deliver and have strong evaluation protocols, but they need assistance in the selection, adaptation, delivery, and evaluation of these interventions. Although it is an implementation laboratory, even the Cooperative Extension is not able to adopt and adapt interventions with fidelity without effective dissemination and intervention testing. More work should be directed to the continued testing, adapting, reporting, and accessibility of evidence-based interventions. This evaluation helps demonstrate ways in which intervention information and adaptations can be conducted, presented, and made available. Generally, we suggest that other organization and integration efforts use RE-AIM and APDER cycles to track changes. Specifically, we demonstrate that the core elements of a behavioral intervention for physical activity promotion among older adults can adapt over time while continuously supporting functional fitness.
